# *Pueraria lobata* polysaccharides alleviate neonatal calf diarrhea by modulating gut microbiota and metabolites

**DOI:** 10.3389/fvets.2022.1024392

**Published:** 2023-01-04

**Authors:** Liuhong Shen, Yu Shen, Liuchao You, Yue Zhang, Zhetong Su, Guangneng Peng, Junliang Deng, Zhicai Zuo, Zhijun Zhong, Zhihua Ren, Shumin Yu, Xiaolan Zong, Yingkun Zhu, Suizhong Cao

**Affiliations:** ^1^The Key Laboratory of Animal Disease and Human Health of Sichuan Province, The Medical Research Center for Cow Disease, College of Veterinary Medicine, Sichuan Agricultural University, Chengdu, Sichuan, China; ^2^Guangxi Innovates Medical Technology Co., Ltd., Lipu, Guangxi, China; ^3^School of Agriculture and Food Science, University College Dublin, Belfield, Ireland

**Keywords:** *Pueraria lobata* polysaccharides, gut microbiota, metabolites, neonatal calf, diarrhea

## Abstract

**Introduction:**

Neonatal calf diarrhea (NCD) is still one of the most critical diseases in calf rearing. Studies have shown that *Pueraria lobata* polysaccharides (PLP) have intense antioxidant and immunomodulatory activity and modulate gut microbiota. This randomized clinical trial aimed to determine the effect of PLP on the neonatal calf with diarrhea.

**Methods:**

In this study, we recorded the fecal score of experimental calves, and calves with fecal scores ≥ 2 were determined as diarrhea and assessed their serum concentrations of inflammatory cytokines and oxidative damage-related indices. Fecal microbiota and metabolomics of diarrheal calves were further investigated.

**Results:**

Results showed that treatment with PLP decreased the fecal score of diarrheal calves, serum concentrations of IL-1β, TNF-γ, and malondialdehyde, and also elevated the level of superoxide dismutase. In addition, PLP treatment altered the gut microbiota, significantly increased the relative abundances of beneficial bacteria, including the phyla Bacteroidetes and Actinobacteria, the genus Collinsella, Megamonas, and Bifidobacterium; decreased the relative abundances of pathogenetic or diarrhea related bacteria, such as Proteobacteria, Fusobacteria, Clostridium_sensu_stricto_1, and Escherichia_Shigella. Moreover, PLP can increase the fecal concentrations of isobutyric acid, propionic acid, and pantothenate; lower the levels of PC [18:0/18:1(9Z)], arachidonic acid, and docosahexaenoic acid.

**Discussion:**

Thus, the results suggested that the PLP may perform the therapeutic activity via alleviating intestinal inflammation and regulating gut microbiota, avoiding further dysbiosis to restore the metabolism of gut microbiota, and finally promoting the recovery of diarrhea. The change further mitigated intestinal inflammation and oxidative damage in diarrheal calves. This indicated that PLP might be a promising treatment to attenuate diarrhea in neonatal calves.

## 1. Introduction

Neonatal calf diarrhea (NCD) has long been a problem in the dairy industry and caused severe economic losses, which even affect morbidity and mortality within the first month of life, and calves' later productive and reproductive performance (1, 2). Since intestinal development in young animals is immature, their gut microbiota is highly vulnerable to infections and interferes with their growth (3). Increasing evidence has revealed that the health of animals is closely related to their early life gut microbiota (4). However, a complicated association between infectious and non-infectious factors is all linked to an increased risk of neonatal gut diseases (5). Antibiotics are often used to treat or prevent diarrhea in ruminants that are known to cause antibiotic-resistant bacteria (6), and more importantly, antibiotic therapy of diarrheic calves leads to recurrence of severe diarrhea and aggravates intestinal dysbiosis (7, 8). Overall, NCD is a multifactorial and challenging disease. Therefore, alternative treatments for diarrhea, with fewer side effects, minimized residues, and resistance has become a critical spot for the sustainable dairy industry.

*Pueraria lobata* is one of the traditional Chinese medicinal herbs which has been widely utilized (9) and contains biologically active substances, including isoflavones, polysaccharides, amino acids, and terpenoids (10). Polysaccharides have been reported as a prebiotic with functions of promoting the growth of beneficial gut bacteria, reducing intestinal pathogens through competitive inhibition, and sustaining the homeostasis of the intestinal environment and host health (11, 12). Studies have proved that the *Pueraria lobata* polysaccharide (PLP) has strong antioxidant activity and immunomodulatory activity *in vitro* (13) and modulates gut microbiota (14). However, few researchers have focused on the effects of PLP on neonatal calf growth. Moreover, the impact of polysaccharides on neonatal calf diarrhea might be closely related to the gut microbiota. It has been proved that the initial development of gut microbiota has a long-term physiological effect on the host (12). However, the intestinal micro-ecology of the calf is immature, and various pathogenic bacteria and their metabolites could initiate the early phase of diarrhea infection (15). Although the traditional focus on calf diarrhea has been on direct pathogens like *Escherichia coli, Salmonella*, and *C. perfrigens* (16), increasing studies suggest that gut microbiota is significantly associated with the health status of animals (17, 18). Previous research has found that the reduction of Bacteroidetes and elevation of Proteobacteria were observed in neonatal diarrhea calves compared with healthy calves (19). A healthy gut microbiota would support its host by inhibiting potential pathogenic bacteria and the maturation of the intestinal immune system by providing beneficial metabolites (20), like short-chain fatty acids (SCFAs), which are essential for intestinal health (21). Thus, the changes in the gut microbiota and their metabolites during the treatment should be emphasized when developing novel NCD therapies.

Hence, this study investigated the impact of oral PLP administration on fecal microbiota, metabolites profiles, and serum inflammatory indices on NCD calves to investigate the non-antibiotic therapy for NCD further.

## 2. Materials and methods

### 2.1. Preparation of PLP

PLP was provided by Guangxi Innovate Medical Technology Co., Ltd. and identified by the National Engineering Research Center of Traditional Chinese Medicine Solid Preparation Manufacturing Technology from Jiangxi University of Traditional Chinese Medicine. The average Mw of PLP, with 50.00% purity, was 1.09 × 10^5^ Da, and the monosaccharide residual was glucose (22).

### 2.2. Animals

The study was conducted on an intensively managed dairy farm in Sichuan, China. Dairy calves were offered 4 L colostrum within 2 h after birth, then housed individually with bedding material to avoid physical contact with each other. After the first day of life, the calves were fed milk from a bucket twice a day at 8:00 and 16:00, with free access to concentrate and water for the next 7 days.

Calves without any previous antibiotic treatment and with a fecal score of 2 (runny, spreads easily) or 3 (liquid, devoid of solid material) were defined as NCD calves and enrolled in this study (23); the NCD was considered to end when their fecal score was ≤ 1 for two consecutive days (24). Investigate the fecal score of enrolled calves for seven consecutive days. The day of enrollment was defined as 0 day.

According to the criterion, twelve NCD calves (7–8-day ages and 45–55 kg weights) were selected from fifty neonatal calves and orally administered PLP (400 mg/kg BW) QD for five consecutive days. Each calf was administered using an oral dispenser to ensure a consistent dose. The dosage of PLP was conducted by referencing a similar study with some modifications (22). Meanwhile, twelve age-matched healthy calves with a fecal score of 0 (normal consistency to feces) or 1 (semiformed or pasty feces) were selected as the control group (23).

Fecal samples and blood samples were collected from the rectum and tail of NCD calves (DS) and calves in the control group (HS) on the 0 day; then collected fecal samples and blood samples from NCD calves treated with PLP (TS) on the 7 days of the trial. Blood samples were collected *via* the caudal vein using the non-anticoagulation tube, centrifuged at 1,500 × *g* for 10 min at room temperature, and then collected serum. Immediately freeze all samples at −80°C until the end of the trial. After the sample collection, feces samples were sent to Shanghai Applied Protein Technology Co., Ltd. (Shanghai, China) for analysis.

### 2.3. Serum analysis

Serum IL-1β, TNF-α, malondialdehyde (MDA), and superoxide dismutase (SOD) were measured using commercially available test kits from Shanghai Enzyme-linked Biotechnology Co., Ltd., China. All testing procedures were strictly following the manufacturer's instructions.

### 2.4. 16S rDNA gene sequencing for analysis of gut microbiota

Microbial genomic DNA was extracted using the DNA kit and detected using agarose gel electrophoresis. The extracted DNA from each sample was amplified at the bacterial 16S rDNA V3–V4 region using specific primers: 341F (5′-CCTACGGGRBGCASCAG-3′) and 806R (5′-GGACTACNNGGGTATCTAAT-3′). The PCR products were purified by AxyPrep DNA Gel Extraction Kit (Axygen Biosciences, USA). Generated the sequencing libraries using the NEB Next^®^Ultra™DNA Library Prep Kit for Illumina (NEB, USA) and added index codes. DNA libraries were loaded and sequenced on an Illumina NovaSeq 6000 platform. Reads were assembled using FLASH and analyzed using QIIME. Sequences with more than 97% similarity were assigned to the same operational taxonomic units. Generated the Shannon-index curves and calculated the Chao1 and Shannon indices using Qiime, then explored the β-diversity of the microbial communities *via* non-metric multidimensional scaling (NMDS) based on weighted uniFrac distances. The linear discriminant analysis (LDA) effect size (LEfSe) method analyzed the biomarkers. The data was completed using the Wekemo Bioincloud (https://www.bioincloud.tech/task-meta).

### 2.5. Metabolomics analysis of fecal samples

Methanol/acetonitrile/H_2_O (2:2:1, v/v/v) were added to fecal sample for metabolite extraction. Analysis was performed using an ultra-high pressure liquid chromatography system (1290 Infinity LC, Agilent Technologies) coupled to a quadrupole time-of-flight (AB Sciex Triple TOF 6600) mass spectrometer (UHPLC-Q-TOF/MS). Compound identification of metabolites was performed by comparing accuracy *m/z* value (< 25 ppm) and MS/MS spectra with an in-house database established with available authentic standards. The processed data were subjected to multivariate data analysis by the *R* package. Metabolites with variable importance for the projection (VIP) values higher than 1 were further applied to Student's *t*-test at the univariate level to measure the significance of each metabolite. The *P*-value < 0.05 was considered statistically significant. Metabolites with log_2_ (fold change) (log_2_FC) higher than 1 or lower than 0 were determined as upregulated or downregulated, respectively.

### 2.6. Correlation analysis

The correlation analysis was conducted using the OmicStudio tools (https://www.omicstudio.cn/tool). Spearman's correlation analysis was performed for the peak areas of the selected metabolites, bacteria, and biochemical indices. A co-expression network was constructed using Cytoscape 3.9.1 software. *P*-values ≤ 0.05 signified a significant correlation. The correlation coefficient range (*r*) was from −1 to 1, and *r* > 0 or < 0 represented a positive or negative correlation.

### 2.7. Statistical analysis

The results of biological data and fecal score were expressed as mean ± SEM and analyzed *via* SPSS 26.0 software. The student's *t*-test analyzed the differences between the two groups. Multiple group comparisons were analyzed using one-way ANOVA. The statistical significance is determined if *P* < 0.05. The LEfSe method and the Kruskal-Wallis (KW) rank sum test were performed to identify features characterizing significant differences between two assigned classes. A value of LDA >3.0 was considered statistically significant. Analysis of differential expression of metabolites was performed using *P*-values and VIP values conducted by orthogonal partial least squares discrimination analysis (OPLS-DA).

## 3. Results

### 3.1. Efficacy of PLP in alleviating diarrhea

**Figure 1:**
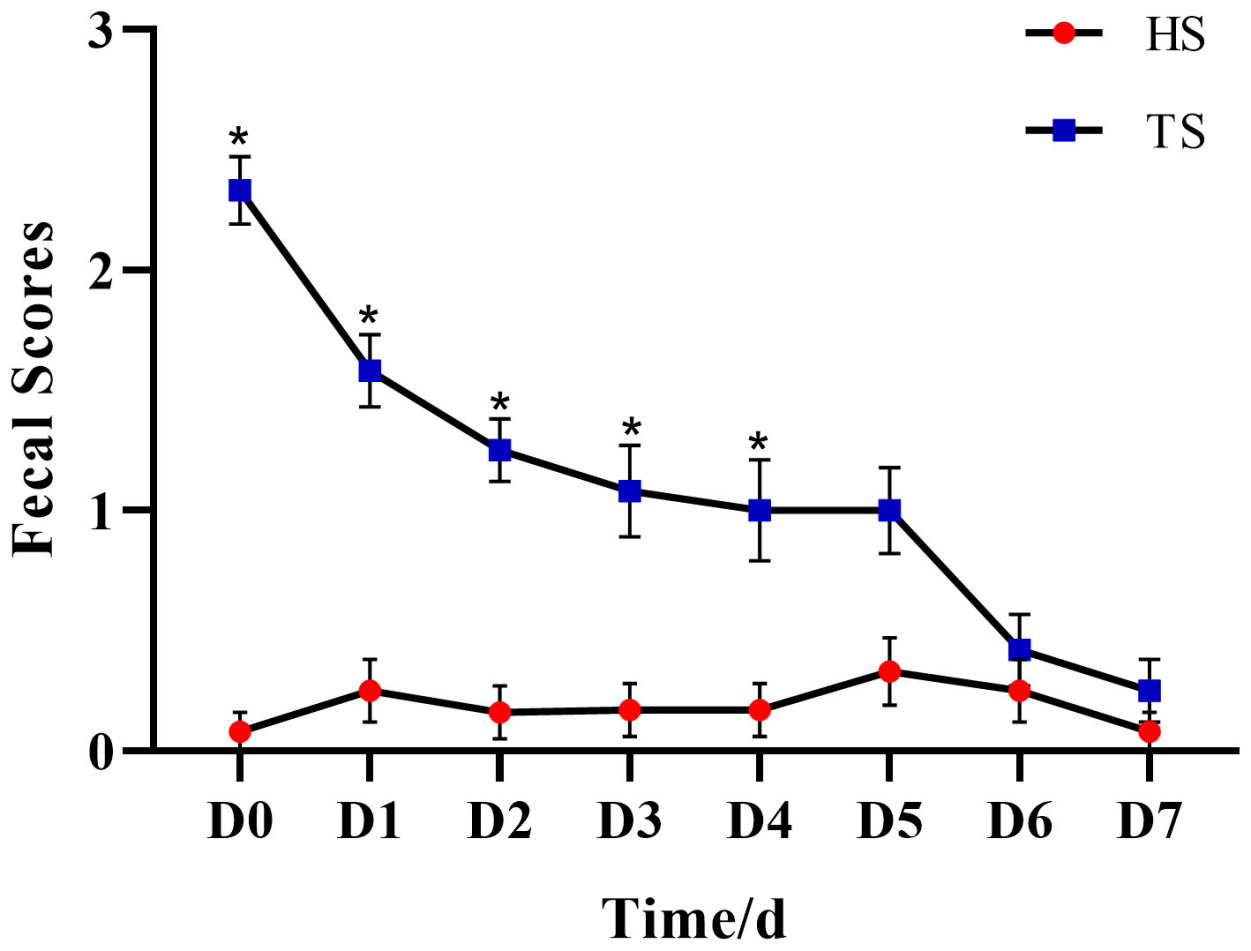
The effect of *Pueraria lobata* polysaccharide (PLP) on fecal scores of calves. *P < 0.05 HS vs. TS, D0 means diarrheal calf without PLP treated (DS). HS, healthy calf fecal sample; TS, PLP treatment diarrheal calf fecal sample.

The anti-diarrhea efficacy of PLP was performed by the conditions of feces (Supplementary Figure 1). Figure 1 and Supplementary Table 1 show that the fecal score gradually decreased with the treatment time. By day 5, there was no significant difference between HS and TS groups (*P* = 0.082), which suggested that PLP treatment alleviated diarrheal symptoms.

### 3.2. PLP effect on oxidative stress, immune, and inflammatory indices

**Figure 2:**
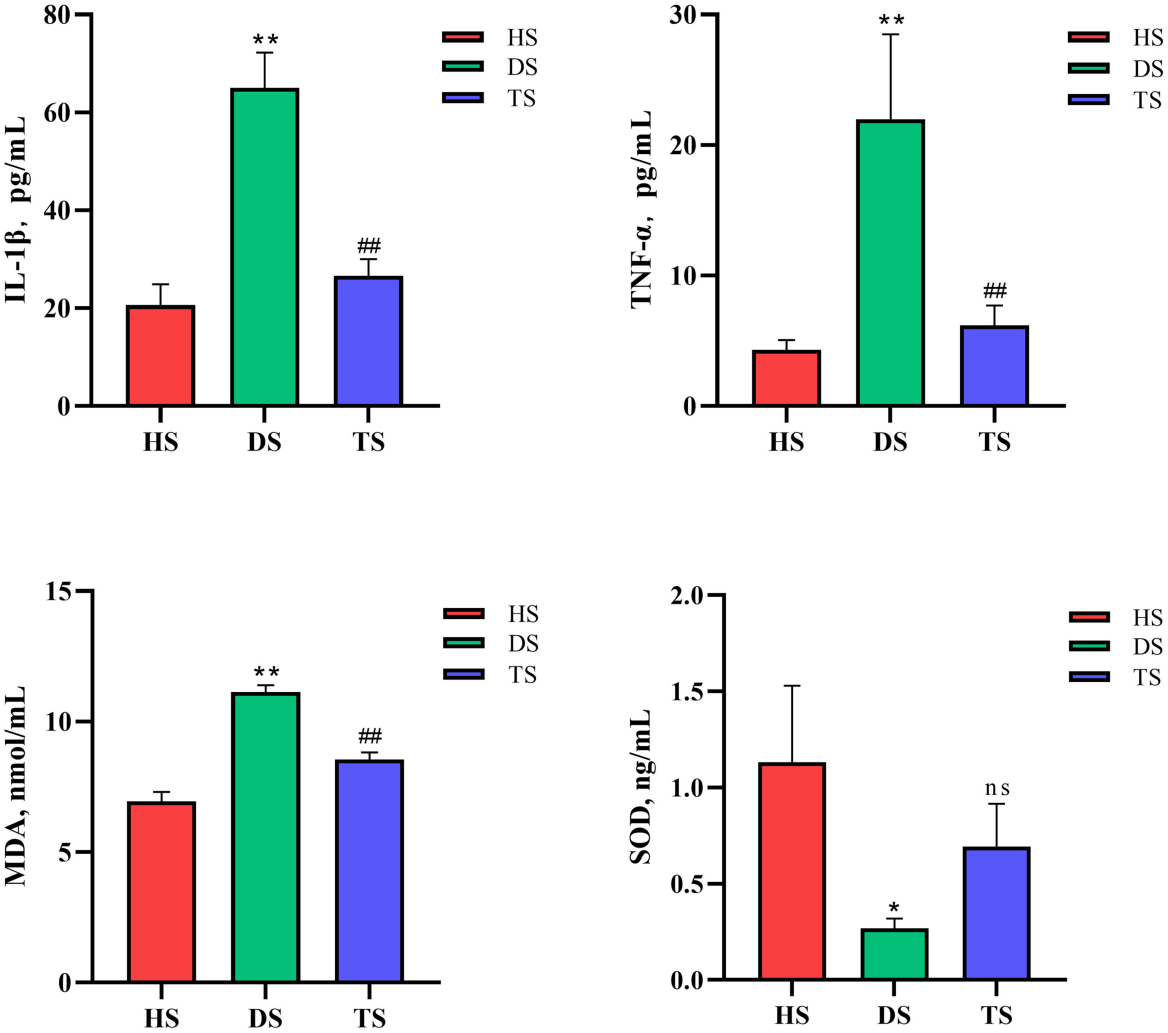
Effect of *Pueraria lobata* polysaccharide (PLP) on the oxidative stress level and immune inflammatory cytokines. Expression of the IL-1β, TNF-α, malondialdehyde (MDA) and superoxide dismutase (SOD). Each column represents a sample. Data are expressed as mean ± SEM, SEM means standard error of the mean, *P < 0.05, **P < 0.01 DS vs. HS; ^##^*P* < 0.01 TS vs. DS. ns, no significant. HS, healthy calf fecal sample; DS, diarrheal calf fecal sample; TS, PLP treatment diarrheal calf fecal sample.

NCD calves showed significantly higher serum IL-1β and TNF-α concentrations compared with the healthy calves (*P* < 0.01, HS vs. DS), while the PLP therapy significantly alleviated the rising of IL-1β and TNF-α (*P* < 0.01, TS vs. DS). Serum MDA concentration was significantly increased in the diarrheal calves compared to the healthy calves (*P* < 0.01), while after treated with PLP in diarrhea calves, which showed reduced activity of MDA compared to the diarrheal calves (*P* < 0.01). Additionally, the expression levels of SOD decreased in the diarrheal calves (*P* = 0.028), which tended to increase after the administration of PLP (*P* = 0.263) (Figure 2).

In conclusion, the PLP effectively reduced oxidative stress levels and immune inflammation in diarrheal calves.

### 3.3. PLP altered the structure of gut microbiota and reversal diarrhea-induced gut microbial dysbiosis

**Figure 3:**
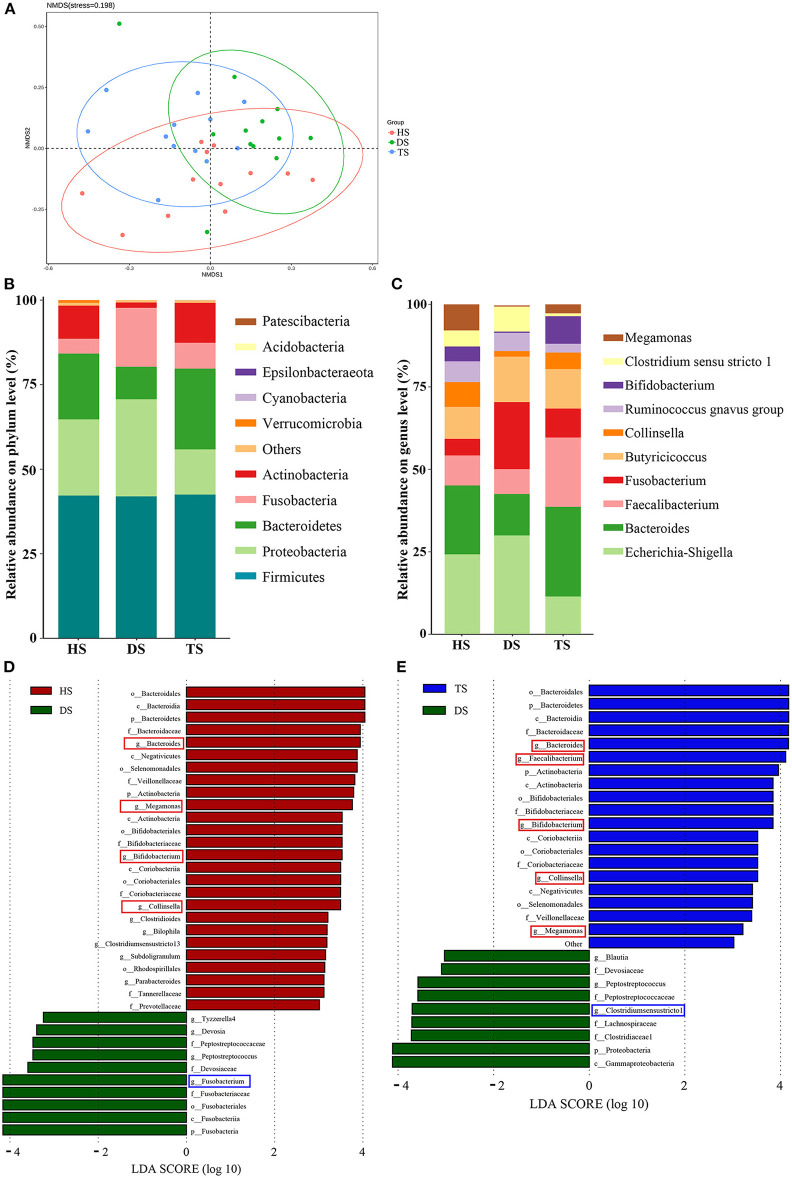
Comparison of the fecal-microbiota structures and distributions in different groups. **(A)** Plots of non-metric multidimensional scaling (NMDS) of 3 groups; **(B, C)** Relative abundance of the fecal microbiota at the phylum and genus level; **(D, E)** Linear discriminant analysis (LDA) among different groups from the linear discriminant analysis. The linear discriminant analysis effect size method identifies the most differentially abundant bacterial taxa, but only taxa meeting an LDA significance threshold of >3.0 are shown. HS, healthy calf fecal sample; DS, diarrheal calf fecal sample; TS, PLP treatment diarrheal calf fecal sample; PLP, Pueraria lobata polysaccharide.

16S rDNA sequencing analysis was conducted to investigate gut microbiota's structural changes. The Shannon-index curve of each sample tended to be flat with the increase in the number of sequences, demonstrating that most diversity was captured (Supplementary Figure S2). For the α-diversity analysis, Chao1 and Shannon indices reflected the community richness and microbiota diversity, respectively. There were no significant differences in Chao1 and Shannon indices among groups HS, DS, and TS (Supplementary Figure S2). Based on weighted UniFrac distances, the NMDS revealed that microbiota structures in the DS group were separated from the HS group (Figure 3A). However, the microbiota structures in the TS group were close to the HS group after PLP administration. It means that PLP could influence the microbiota structures in diarrheal calves.

In the further analysis of gut microbiota at the phylum level, the results indicated that dominant bacteria in each group were composed of Firmicutes, Bacteroidetes, Proteobacteria, Fusobacteria, and Actinobacteria (Figure 3B). Within the healthy calves, Firmicutes (42.21%), Bacteroidetes (19.44%), Proteobacteria (22.51%), Fusobacteria (4.40%), and Actinobacteria (9.78%) were the most abundant. Compared to the healthy calves, diarrhea was associated with proliferated Proteobacteria and Fusobacteria and reduced Bacteroidetes and Actinobacteria, and PLP administration of TS reversed the altering phyla in DS, inhibited Proteobacteria (28.71–13.35%) and Fusobacteria (17.42–7.63%); promoted Bacteroidetes (9.61–23.88%) and Actinobacteria (1.58–11.83%). Finally, among the cross-comparisons of different groups (HS vs. DS and DS vs. TS), the results showed that PLP especially altered four microbial phyla in diarrheal calves: Bacteroidetes, Proteobacteria, Fusobacteria, and Actinobacteria.

At the genus level, the heatmap showed the top 10 genera among all groups (Figure 3C). Compared with the healthy calves, diarrheal calves showed significantly decreased relative abundances of *Bacteroides* and *Megamonas* (*P* < 0.05) and significantly increased *Fusobacterium* (*P* < 0.05). PLP altered the reduction of *Bacteroides* (*P* < 0.01), *Bifidobacterium* (*P* < 0.01) and *Faecalibacterium* (*P* < 0.05) in diarrheal calves and induced significantly abundance decrease in *Clostridium_sensu_stricto_1* (*P* < 0.05) and *Escherichia_Shigella* (*P* < 0.05) (Table 1).

**Table 1:** The relative abundance of top 10 genera during the PLP treatment.

**Relative abundance (%)**	**HS**	**DS**	**TS**
*Bacteroides*	16.99 ± 2.99	9.38 ± 2.26^*^	23.42 ± 2.39^##^
*Escherichia_Shigella*	20.61 ± 5.67	24.02 ± 5.11	9.80 ± 2.22^#^
*Faecalibacterium*	7.36 ± 2.65	6.07 ± 2.70	18.24 ± 5.29^#^
*Butyricicoccus*	8.09 ± 2.32	10.76 ± 2.51	10.41 ± 3.06
*Fusobacterium*	4.40 ± 2.48	17.41 ± 6.10^*^	7.63 ± 4.15
*Bifidobacterium*	3.54 ± 0.75	0.30 ± 0.07	7.18 ± 2.25^##^
*Megamonas*	6.45 ± 2.70	0.48 ± 0.28^*^	2.40 ± 1.20
*Collinsella*	6.16 ± 2.61	1.24 ± 1.03	4.60 ± 3.56
*[Ruminococcus] gnavus group*	5.28 ± 1.12	3.78 ± 1.07	2.29 ± 0.52
*Clostridium_sensu_stricto_1*	4.09 ± 1.96	5.92 ± 2.23	0.75 ± 0.18^#^

All data are presented as mean ± SEM.

^*^P < 0.05, DS vs. HS;

^#^P < 0.05,

^##^P < 0.01 TS vs. DS. HS, healthy calf fecal sample; DS, diarrheal calf fecal sample; TS, PLP treatment diarrheal calf fecal sample.

To find the biomarkers of rectal microflora among various groups, the LEfSe (LDA > 3) method was applied (Figures 3D, E). The results of the HS vs. DS and DS vs. TS overlapped. The detailed analysis at the genus level found that the potential pathogens, *Fusobacterium* and *Clostridium_sensu_stricto_1*, were significantly altered in diarrheal calves, while the potential probiotics, *Collinsella, Megamonas*, and *Bifidobacterium* were considerably increased after PLP treatment. These results suggest that PLP partially restored rectal microbial communities, which could benefit the host.

### 3.4. PLP regulated gut metabolites in NCD and screening for potential biomarkers in NCD

**Figure 4:**
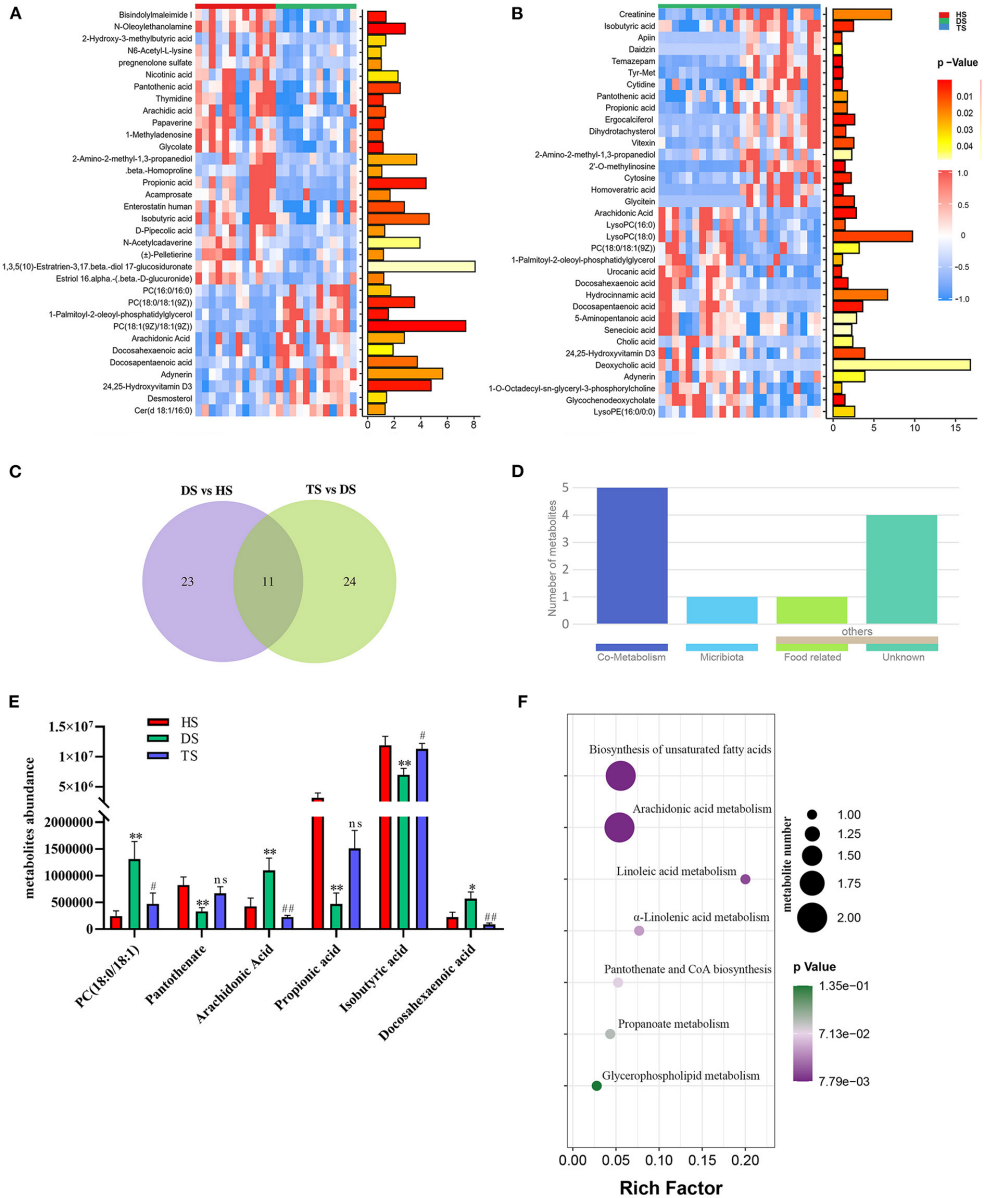
The effect of *Pueraria lobata* polysaccharide (PLP) on diarrheal calf metabolites. **(A)** Heatmap of the 34 notably different metabolites between DS vs. HS; **(B)** Heatmap of the 35 notably altered metabolites between DS vs. TS; **(C)** Veen diagram showed that 11 of the same metabolites were identified in different groups; **(D)** The roles of same metabolites were further analyzed; **(E)** 6 key metabolites concentrations from intestinal contents in different groups; **(F)** The bubble diagrams of metabolic pathways. HS, healthy calf fecal sample; DS, diarrheal calf fecal sample; TS, PLP treatment diarrheal calf fecal sample. *P < 0.05, **P < 0.01 DS vs. HS; ^#^*P* < 0.05, ^##^*P* < 0.01 TS vs. DS; ns, no significant.

The complex interactions between host and gut microbiota are intensely associated with host-microbe metabolic axes. Hence, the untargeted metabolomics analysis on fecal samples by UHPLC Q-TOF/MS. The distinct clustering of metabolites was apparent among the HS, DS, and TS groups in the OPLS-DA (Supplementary Figure S3). The *R*^2^*Y* and *Q*^2^ were used to evaluate the modeling and prediction ability of the OPLS-DA model, respectively (Supplementary Figure S3). There were 34 different metabolites between DS vs. HS group and 35 between DS vs. TS group (Figures 4A, B; Supplementary Tables S2, S3). A Veen diagram showed that 11 common metabolites were identified and partially correlated by PLP treatment (Figure 4C). The mammalian gut microbiota interacts extensively with the host through the co-metabolism of substrates in the intestinal contents. The metabolites were further analyzed for their role using the MetOrigin (http://metorigin.met-bioinformatics.cn/). Results showed that there were 5 common metabolites between host and microbiota, 1 unique metabolite of microbiota, and 5 other metabolites (Figure 4D). In detail, the significant metabolites were isobutyric acid, propionic acid, PC [18:0/18:1(9Z)], arachidonic acid (ARA), docosahexaenoic acid (DHA), and pantothenate (Figure 4E). The KEGG metabolic pathways further retrieved these altered metabolites by MetaboAnalyst (Figure 4F), which are involved in the biosynthesis of unsaturated fatty acids, arachidonic acid metabolism, linoleic acid metabolism, α-linolenic acid metabolism, pantothenate and CoA biosynthesis, propanoate metabolism and glycerophospholipid metabolism.

### 3.5. Correlation analysis between microbes and metabolites

**Figure 5:**
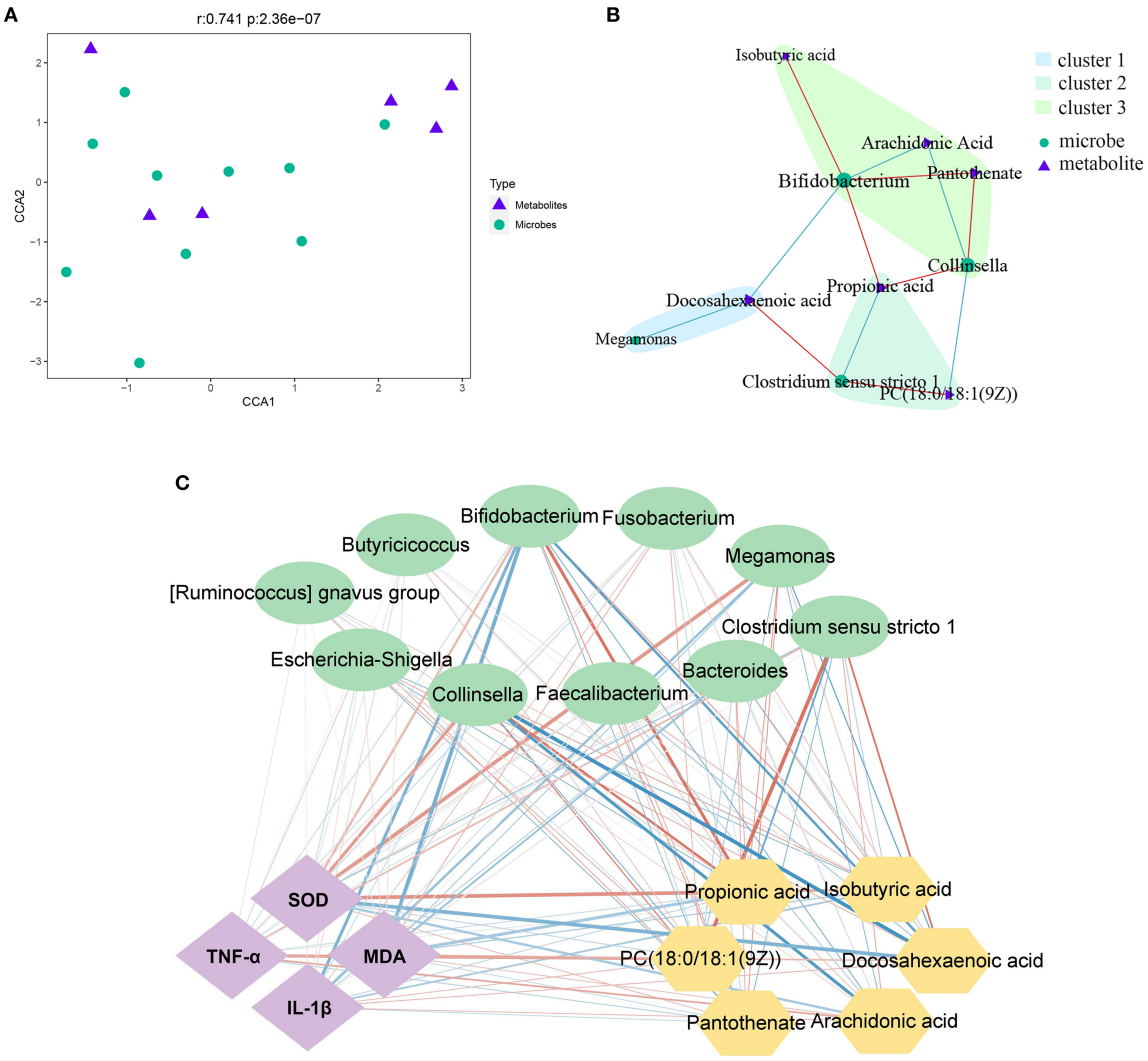
Correlation analysis between microbes and metabolites. **(A, B)** The canonical correlation analysis (CCA) and the pair correlation analysis between metabolites and microbiota. **(C)** The interaction network among phenotype index, bacteria and metabolites; the strength of the interaction is represented by the thickness and depth of the lines. The stronger the interaction, the thicker and darker a line is. The red line represents the positive relationship, and the blue line represents the negative relationship. MDA, malondialdehyde; SOD, superoxide dismutase.

The 16S rDNA sequencing and metabolomics analysis revealed the effect of PLP treatment on diarrheal calves. The correlation analysis showed that there was a significant global correlation between metabolites and microbiota (*r* = 0.741, *P* < 0.001) (Figure 5A). The further pairwise correlation analysis showed that there were 4 highlight microbes linked with the significant metabolites, *Megamonas, Clostridium_sensu_stricto_1, Collinsella*, and *Bifidobacterium* (Figure 5B).

On the other hand, based on phenotype, fecal microbiota at the genus level, and the most differential metabolites, Spearman's correlation analysis identified the relationship upon PLP treatment (Figure 5C). Metabolites such as docosahexaenoic acid, was significant decreased after PLP treatment and showed a strong positive correlation with *Clostridium_sensu_stricto_1* (*r* = 0.611, *P* < 0.01), while negative correlation with *Bifidobacterium* (*r* = −0.625, *P* < 0.01). PC [18:0/18:1(9Z)] had positive correlation with *Clostridium_sensu_stricto_1* (*r* = 0.628, *P* < 0.01), but negative correlation with *Collinsella*. Arachidonic acid was negatively correlated with *Bifidobacterium* (*r* = −0.552, *P* < 0.01) and *Collinsella* (*r* = −0.673, *P* < 0.01). *Bifidobacterium* had positive correlation with isobutyric acid, propionic acid and pantothenate (*r* > 0.5, *P* < 0.01). *Collinsella* also had positive correlation with propionic acid and pantothenate (*r* > 0.5, *P* < 0.01). Specifically, propionic acid was negatively correlated with *Clostridium_sensu_stricto_1* (*r* = −0.576, *P* < 0.01). Based on these results, further analysis to explore the relationship with phenotype. The result showed that *Bifidobacterium* and *Collinsella* were both negatively correlated with MDA (*P* < 0.05), while the metabolites of PC [18:0/18:1(9Z)] positively correlated with MDA (*P* < 0.05). Bifidobacterium was negatively correlated with IL-1β (*P* < 0.01), and DHA was positively correlated with IL-1β (*P* < 0.05). Similarly, propionic acid and isobutyric acid were both positively correlated with SOD (*P* < 0.05), while *Clostridium_sensu_stricto_1* was negatively correlated with SOD (*P* < 0.05). The metabolites of PC [18:0/18:1(9Z)], arachidonic acid, and DHA were positively correlated with TNF-α (*P* < 0.05).

The analysis revealed strong correlations among several specific intestinal bacteria, metabolites, and phenotypes. These data indicated that PLP impacted rectal feces microbiota and metabolites to ameliorate inflammation and oxidative state, further alleviating calf diarrhea.

## 4. Discussion

Diarrhea is a high-risk disease in newborn calves, inducing gut microbiota dysbiosis, metabolic disorders, and even death (25). Therefore, enhancing the gut microbiota may contribute to preventing and alleviating diarrhea (26). A study with rodent models showed that PLP could alleviate inflammation and oxidative stress by restoring gut microbiota dysbiosis (22). Moreover, PLP could relieve colonic pathological changes and gut microbiota dysbiosis caused by antibiotic-associated diarrhea (24). These findings suggest that PLP may be a promising treatment agent for diarrhea-induced intestinal dysbacteriosis and metabolites disorders. The major results of the present study were: (a) PLP administration in calves alleviated inflammation and improved the antioxidant capacity of diarrheal calves. (b) PLP administration inhibited harmful bacteria and modulated the microbiota composition in a probiotic manner. (c) Specific gut-microflora-related metabolites were associated with perturbations in gut microbes.

Cytokines play important roles in inflammatory responses and regulate intestinal barrier integrity (27). Furthermore, the changes in IL-1β and TNF-α are closely related to the development of diarrhea (28). Once the intestinal barrier is compromised, many pathogenic bacteria can colonize the intestinal tract and cause inflammation (29). The Synergy of pathogenic bacterial infection, oxidative stress, and inflammation aggravate the pathological process (30). When the level of oxidative stress increases, SOD plays the role of scavenging free radicals to block lipid peroxidation and MDA production (31). Earlier research found that PLP could promote intestinal barrier integrity and reduce inflammation through the NF-κB signaling pathway (22). In this trial, calves treated with PLP had lower fecal scores, and IL-1β and TNF-α decreased in clinical cure calves compared to the diarrheic ones. Moreover, diarrheic calves treated with PLP could recover the antioxidant capacity, that is, the lower level of MDA and a higher level of SOD in clinical cure calves. It suggested that the PLP had a beneficial effect on inhibiting the inflammatory response and improving the redox state in diarrheic calves.

The gut microbiota of infant animals was not mature, which was more likely to occur diarrhea and exacerbated intestinal dysfunction (3, 32). Detecting gut microbiota is necessary to determine the effectiveness of PLP for calf diarrhea. Consistent with the previous research (16, 33), our study found the phylum Proteobacteria as the most dominant bacteria in healthy calves. However, a widespread addition of the phylum Proteobacteria is a marker for microbial community dysbiosis and a potential diagnostic criterion for disease (34) *via* triggering intensive inflammatory responses by producing lipopolysaccharide (35), disturbing the gut microbiota, damaging the intestinal barrier, and induce the inflammatory response (36). The relative abundance of Proteobacteria in the cure calves (13.35%) was lower than that in the diarrheic calves (28.71%), implying that PLP may inhibit the reproduction of Proteobacteria, a harmful bacterium.

Furthermore, proliferated Fusobacteria in diarrheal calves could invade the intestinal epithelial barrier, inducing diarrhea symptoms (37, 38). The loss of microbial richness was most associated with the over-proliferated *Fusobacterium* (39). In this study, the relative abundance of Fusobacteria decreased in the cure calves (7.63%) compared to the diarrheic calves (17.42%) after PLP treatment. Bacteroidetes and Actinobacteria were beneficial bacteria phyla that can positively contribute to maintaining intestinal homeostasis (40, 41). Moreover, Bacteroidetes species generally produce butyrate, a colonic fermentation product that helps maintain a healthy gut (42). PLP treatment promoted the growth of Bacteroidetes and Actinobacteria in diarrheic calves, indicating that PLP could protect the intestinal tract by reducing pathogenic bacteria and increasing beneficial microbes. At the genus level, the analysis showed that PLP treatment decreased the levels of pathogens, *Clostridium_sensu_stricto_1*, and *Escherichia_Shigella*, in diarrheal calves. Previous studies indicated that *Clostridium_sensu_stricto_1* plays a vital role in developing ulcerative colitis and promotes inflammatory response (43, 44). In the present study, we found that the relative abundance of *Clostridium_sensu_stricto_1* negatively correlated with the level of SOD.

On the other hand, *Escherichia_Shigella* was widely accepted as the primary pathogen to cause bovine diarrhea (45). We speculated that PLP restored the ecological balance of gut microbiota by inhibiting the proliferation of *Clostridium_sensu_stricto_1* and *Escherichia_Shigella* in diarrheic calves. Furthermore, LEfSe analysis identified enrichment of *Bifidobacterium* in the cure calves compared with the diarrheic calves. *Bifidobacterium* produces SCFAs and stimulates the growth of butyrate-producing bacteria (46, 47). *Bifidobacterium* also prevents gastrointestinal infections by the elevated colonization of epithelial cells (47). In other research, inulin-type fructans improved the gut microbiota of mice, increased the abundance of *Bifidobacterium*, and reduced the levels of *Clostridium* and *Escherichia_Shigella* (48). *Collinsella* can metabolize carbohydrates, and cooperation with *Bifidobacterium* modifies the host's bile acids, which modulates the virulence and pathogenicity of enteric pathogens (49). Thus, the reduced pathogenic bacteria and increased beneficial bacteria colonization in the intestinal can be considered one of the advantages of diarrheic calves treated with PLP.

As a carbon source for the gut microbiota, polysaccharides can promote the growth of gut microbiota, which, in turn, ferment polysaccharides into SCFAs (50), which play an essential role in mucosal integrity and immune response (51). Propionic acid exerts immunomodulatory properties by inducing regulatory *T* cell expression (52). Moreover, fecal isobutyric acid concentration was significantly lower in diarrheal calves (53). Through correlation analysis of the bacteria and metabolites, we found that propionic acid and isobutyric acid were significantly positively correlated with *Collinsella* and *Bifidobacterium*. Furthermore, we found that PLP significantly altered the reduction of the content of isobutyric acid and propionic acid in diarrheal calves. We could further demonstrate that PLP affects gut microbiota, particularly raising SCFAs and producing bacteria abundance. The present research showed that the levels of PC [18:0/18:1(9Z)] and ARA were lower after PLP treatment in calves with diarrhea. The present study found that PCs belong to the glycerophosphate group, mainly involved in glycerophospholipid metabolism. The increased PC content suggests that oxidative stress response was evoked (54). The damaged membranes can release ARA to promote ROS, thereby aggravating oxidative stress (55). Therefore, the PLP inhibited oxidative stress and immune-inflammatory in diarrheal calves, related to the restored metabolism of glycerophospholipid, arachidonic acid, and linoleic acid. DHA, a member of omega-3 PUFAs, ameliorated inflammation and improved wound healing (56). However, we found that the level of DHA decreased in cure calves compared with diarrheic calves. We speculated that inflammation response gradually fades in cured calves, which is closely related to the decrease of DHA. But we need further study to be conclusive. Pantothenate is the precursor for CoA synthesis, an essential cofactor for a broad range of functions within all cells, such as the tricarboxylic acid cycle and fatty acid synthesis (57). PLP treatment increased the level of pantothenate, which also positively correlated with the growth of intestinal probiotics. These results were compared well with the alteration of gut microbes, which further supported that PLP ameliorates diarrhea by modulating specific gut-microflora-related metabolites. Overall, PLP supplementation attenuated diarrhea which may be associated with the probiotic improving ability, organic acid production, and immunomodulatory activity caused by PLP fermentation and selective utilization of bacteria in the gut.

## 5. Conclusions

The study revealed that the gut microbiota in diarrheal calves undergoes striking changes, characterized by an increased abundance of pathogenic bacteria and metabolic disturbance in diarrheal calves. Conversely, PLP treatment lowered fecal scores, inhibited the inflammatory response, and improved the redox state to attenuate the diarrhea symptom of calves. These impacts were mainly performed by altering the gut microbiome and promoting SCFA production. Further, PLP administration also restored host metabolism by reducing the serum levels of proinflammatory mediators, PC [18:0/18:1(9Z)], and ARA. PLP could become a promising non-antibiotic treatment for neonatal calf diarrhea. How those PLP-modulated microbial communities affect calves' health and productivity in their later life deserves further investigation.

## Data availability statement

The datasets presented in this study can be found in online repositories. The names of the repository/repositories and accession number(s) can be found in the article/Supplementary material.

## Ethics statement

Sample collection was performed in strict accordance with the guidelines of the Care and Use of Laboratory Animals of China and all procedures were approved by the Animal Care and Use Committee of Sichuan Agricultural University (No. 2013-028).

## Author contributions

LS, YS, YZha, and LY designed the study. ZS, GP, ZZu, ZZh, and JD conducted the experiment. SY and LS performed lab analysis and wrote the manuscript. ZR, SY, and XZ performed statistics and analyzed the data. YZhu and SC revised the article. All authors carefully read and approved the final revision of the manuscript.
