# Supplementary material for: Pueraria lobata polysaccharides alleviate neonatal calf diarrhea by modulating gut microbiota and metabolites
Source: Front Vet Sci. 2023 Jan 4;9:1024392. doi: 10.3389/fvets.2022.1024392 (PMC9845628; doi:10.3389/fvets.2022.1024392)
Supplement: Supplementary file 1 [file Data_Sheet_1.docx]

Supplementary Material

## Supplementary Figures

| Day | HS | TS |
| --- | --- | --- |
| D0 | 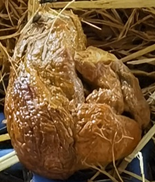 | 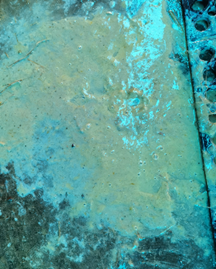 |
| D3 | 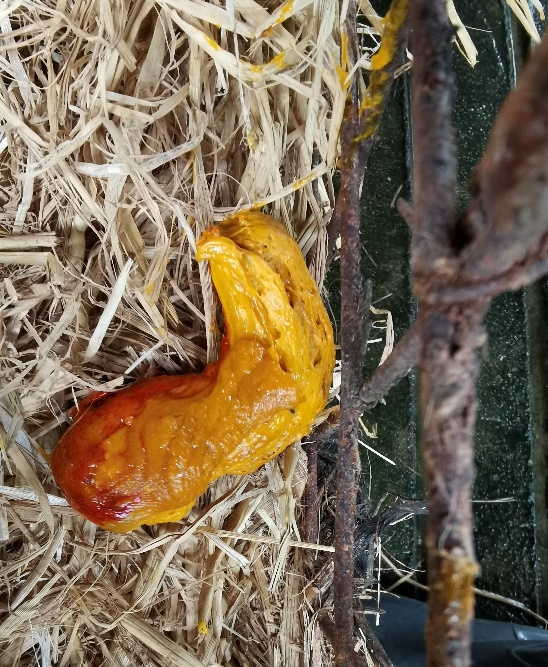 | 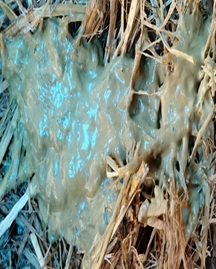 |
| D5 | 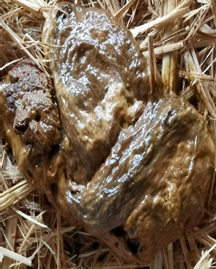 | 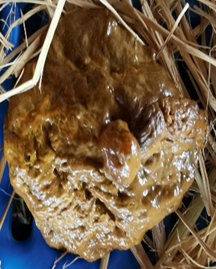 |
| D7 | 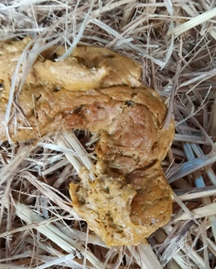 | 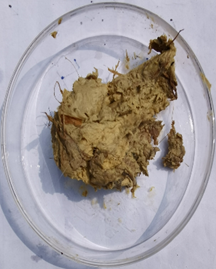 |

**Supplementary Figure 1.** State of feces during Pueraria lobata polysaccharides (PLP) treatment. Results showed that the diarrheal symptoms of calves were alleviated with PLP treatment. D0 means diarrheal calf without PLP treated. HS=healthy calf; TS=diarrheal calf with PLP treated.


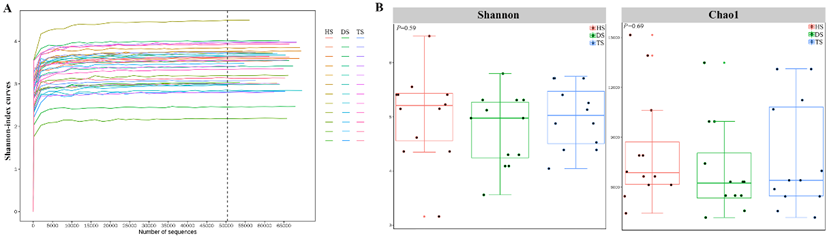


**Supplementary Figure 2.** (A) Shannon-index curve of the HS, DS and TS groups; (B) The α-diversity of different groups by Chao1 and Shannon indices. Data were mean ± SEM. P-values were determined using the nonparametric Kruskal-Wallis test. HS = healthy calf fecal sample; DS = diarrheal calf fecal sample; TS = PLP treatment diarrheal calf fecal sample


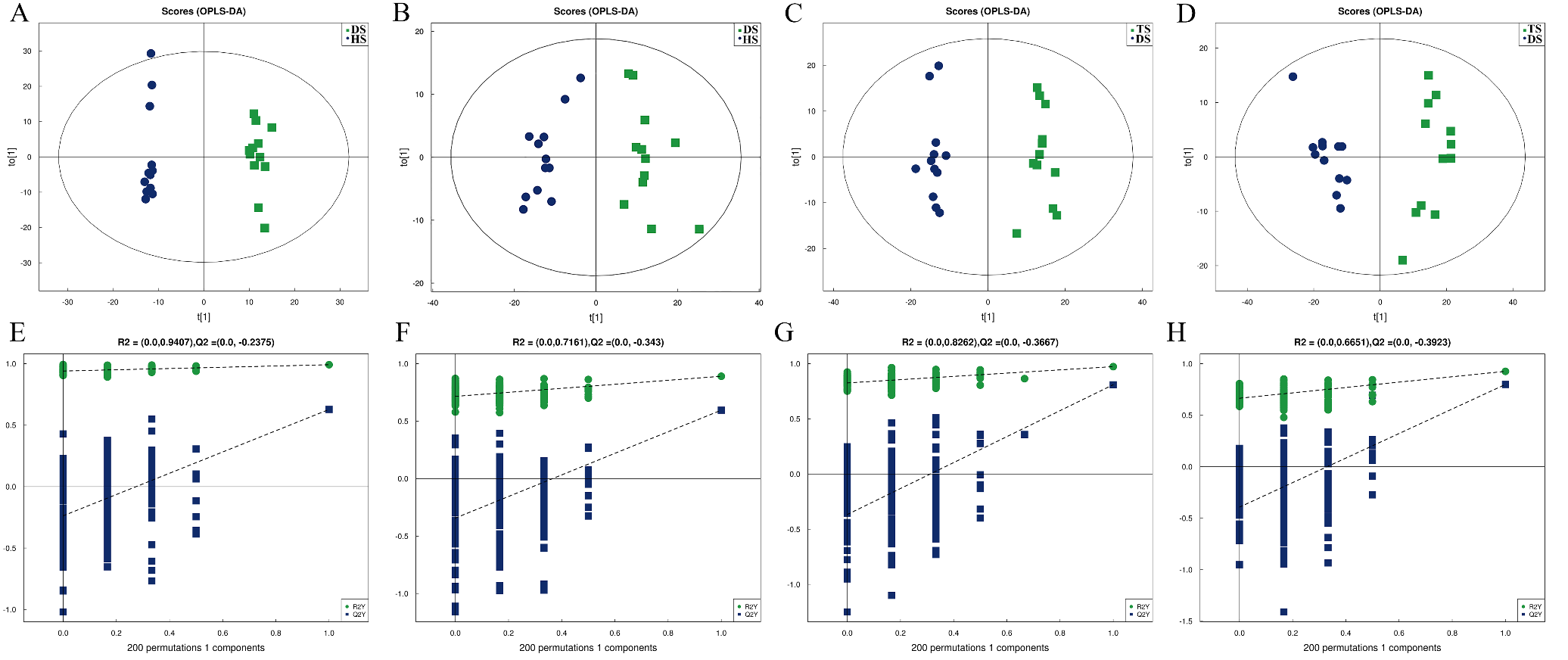


**Supplementary Figure 3.** (A), (E) and (C), (G) Orthogonal partial least square discriminant analysis (OPLS-DA) of scores and permutation test plots for the DS vs HS and TS vs DS samples analyzed in the positive ion mode, respectively. (B), (F) and (D), (H) Orthogonal partial least square discriminant analysis of scores and permutation test plots for the DS vs HS and TS vs DS samples analyzed in the negative ion mode, respectively. t[1] = first principal component. to[1] = second orthogonal component. The intercept limit of Q^2^, calculated by regression line, is the plot of Q^2^ from permutation test in the OPLS-DA model. HS = healthy calf fecal sample; DS = diarrheal calf fecal sample; TS = PLP treatment diarrheal calf fecal sample.

# Supplementary Tables

**Table S1.** The effect of *Pueraria lobata* polysaccharides (PLP) on fecal scores of calves

| Time | Group | | *P*-value |
| --- | --- | --- | --- |
|  | HS | TS |  |
| D0 | 0.08±0.08 | 2.33±0.14^a^ | <0.001 |
| D1 | 0.25±0.13 | 1.58±0.15^b^ | <0.001 |
| D2 | 0.16±0.11 | 1.25±0.13^bc^ | <0.001 |
| D3 | 0.17±0.11 | 1.08±0.19^c^ | <0.001 |
| D4 | 0.17±0.11 | 1.00±0.21^c^ | 0.002 |
| D5 | 0.33±0.14 | 1.00±0.18^c^ | 0.082 |
| D6 | 0.25±0.13 | 0.42±0.15^d^ | 0.409 |
| D7 | 0.08±0.08 | 0.25±0.13^d^ | 0.294 |

^1^All data are presented as mean ± SEM.

^2^The different subscript letter in the same column represents significant difference (*P* < 0.05), the same letter represents insignificant difference (*P* > 0.05).

^3^D0 means diarrheal calf without PLP treated. HS=healthy calf; TS=diarrheal calf with PLP treated.

**Table S2. Differential metabolites identified of DS vs HS in the positive or negative mode**

| **No.** | **Identification** | ***m/z*** | **RT(s)** | **VIP** | **Fold change** | **p-value** | **adduct** |
| --- | --- | --- | --- | --- | --- | --- | --- |
| 1 | N-Oleoylethanolamine | 326.304 | 36.049 | 2.820039 | 0.394339 | 0.000317 | (M+H)+ |
| 2 | PC(18:1(9Z)/18:1(9Z)) | 786.5986 | 148.361 | 7.379876 | 6.709015 | 0.000636 | (M+H)+ |
| 3 | 24,25-Hydroxyvitamin D3 | 381.3135 | 99.861 | 4.76962 | 2.791994 | 0.00292 | (M+H-2H2O)+ |
| 4 | Papaverine | 357.1757 | 200.1785 | 1.228778 | 0.273416 | 0.004579 | (M+NH4)+ |
| 5 | Thymidine | 243.0959 | 103.2575 | 1.155154 | 0.361954 | 0.004739 | (M+H)+ |
| 6 | PC(18:0/18:1(9Z)) | 810.596 | 146.074 | 3.527916 | 5.386628 | 0.004891 | (M+Na)+ |
| 7 | Pantothenate | 220.1172 | 276.944 | 2.445079 | 0.405721 | 0.008279 | (M+H)+ |
| 8 | 1-Methyladenosine | 282.1177 | 262.194 | 1.101817 | 0.340739 | 0.012517 | (M+H)+ |
| 9 | Pelletierine | 142.1212 | 279.064 | 1.184973 | 0.339807 | 0.021294 | (M+H)+ |
| 10 | D-Pipecolinic acid | 130.0851 | 317.321 | 1.275741 | 0.186021 | 0.022048 | (M+H)+ |
| 11 | Cer(d 18:1/16:0) | 538.5154 | 34.57 | 1.308176 | 1.51674 | 0.023 | (M+H)+ |
| 12 | .beta.-Homoproline | 130.085 | 78.202 | 1.061004 | 0.27206 | 0.023309 | (M+H)+ |
| 13 | Arachidonic Acid | 305.2458 | 36.35 | 2.756612 | 2.568655 | 0.024504 | (M+H)+ |
| 14 | 2-Amino-2-methyl-1,3-propanediol | 70.06492 | 323.985 | 3.68337 | 0.219075 | 0.024523 | (M+H-2H2O)+ |
| 15 | PC(16:0/16:0) | 756.5498 | 150.032 | 1.738577 | 3.526691 | 0.028416 | (M+Na)+ |
| 16 | N6-Acetyl-L-lysine | 189.1218 | 406.26 | 1.008306 | 0.386971 | 0.029669 | (M+H)+ |
| 17 | Desmosterol | 367.3327 | 111 | 1.415116 | 3.404863 | 0.033008 | (M+H-H2O)+ |
| 18 | Nicotinate | 124.0381 | 221.9065 | 2.276412 | 0.413734 | 0.034063 | (M+H)+ |
| 19 | N-Acetylcadaverine | 145.1327 | 346.568 | 3.934137 | 0.249666 | 0.044703 | (M+H)+ |
| 20 | Glycolate | 75.00848 | 305.366 | 1.161412 | 0.31042 | 0.001633 | (M-H)- |
| 21 | Bisindolylmaleimide I | 411.1806 | 32.1005 | 1.375562 | 0.338776 | 0.001852 | (M-H)- |
| 22 | 1-Palmitoyl-2-oleoyl-phosphatidylglycerol | 747.5129 | 39.3385 | 1.553457 | 6.411898 | 0.002131 | (M-H)- |
| 23 | Propionic acid | 73.0297 | 171.381 | 4.389979 | 0.148493 | 0.002695 | (M-H)- |
| 24 | Enterostatin human | 495.2746 | 59.095 | 2.757123 | 0.466243 | 0.010475 | (M-H)- |
| 25 | Arachidic acid | 311.2939 | 38.4875 | 1.332585 | 0.411403 | 0.011311 | (M-H)- |
| 26 | Isobutyric acid | 87.04579 | 119.756 | 4.620276 | 0.586055 | 0.012814 | (M-H)- |
| 27 | Docosapentaenoic acid | 329.2466 | 40.018 | 3.733835 | 2.50354 | 0.015888 | (M-H)- |
| 28 | Estriol 16.alpha.-(.beta.-D-glucuronide) | 463.194 | 126.317 | 1.206853 | 0.324641 | 0.018815 | (M-H)- |
| 29 | Acamprosate | 180.0322 | 124.627 | 1.677938 | 0.269528 | 0.019355 | (M-H)- |
| 30 | pregnenolone sulfate | 395.1857 | 28.171 | 1.022142 | 0.32417 | 0.021608 | (M-H)- |
| 31 | Adynerin | 515.3007 | 52.1985 | 5.644513 | 2.536602 | 0.022668 | (M-H)- |
| 32 | 2-Hydroxy-3-methylbutyric acid | 117.0547 | 182.863 | 1.360205 | 0.243795 | 0.03108 | (M-H)- |
| 33 | Docosahexaenoic acid | 327.2299 | 42.9895 | 1.907181 | 2.56107 | 0.037085 | (M-H)- |
| 34 | 1,3,5(10)-Estratrien-3,17.beta.-diol 17-glucosiduronate | 447.2051 | 91.1345 | 8.082374 | 0.333739 | 0.04822 | (M-H)- |

^1^The adduct column showed the positive or negative mode. *m/z* = mass-to-charge ratio; RT = retention time; VIP = variable importance in projection. D0 means diarrheal calf without PLP treated. HS=healthy calf; TS=diarrheal calf with PLP treated. The same below.

**Table S3.** Differential metabolites identified of TS vs DS in the positive or negative mode

| **No.** | **Identification** | ***m/z*** | **RT(s)** | **VIP** | **Fold change** | **p-value** | **adduct** |
| --- | --- | --- | --- | --- | --- | --- | --- |
| 1 | 2'-O-methylinosine | 343.1197 | 38.372 | 1.462974 | 29.24788 | 2.08E-06 | (M+CH3COO+2H)+ |
| 2 | Tyr-Met | 295.1157 | 36.7695 | 1.185426 | 4.1654 | 4.42E-05 | (M+H-H2O)+ |
| 3 | Temazepam | 301.0687 | 40.625 | 1.128833 | 11.88314 | 0.000189 | (M+H)+ |
| 4 | Glycochenodeoxycholate | 467.344 | 227.424 | 1.446625 | 0.308413 | 0.000298 | (M+NH4)+ |
| 5 | Arachidonic Acid | 305.2458 | 36.35 | 2.840118 | 0.205471 | 0.001164 | (M+H)+ |
| 6 | Cytidine | 226.0807 | 186.257 | 1.100641 | 2.432792 | 0.001869 | (M+H-H2O)+ |
| 7 | Ergocalciferol | 457.3656 | 72.299 | 2.642823 | 22.9069 | 0.001956 | (M+CH3COO+2H)+ |
| 8 | Urocanic acid | 139.0489 | 296.592 | 1.021301 | 0.279464 | 0.003143 | (M+H)+ |
| 9 | Cytosine | 112.0494 | 210.3755 | 2.210064 | 3.592775 | 0.005111 | (M+H)+ |
| 10 | Glycitein | 285.0749 | 24.64 | 2.599895 | 171.7217 | 0.006158 | (M+H)+ |
| 11 | Dihydrotachysterol | 421.3434 | 72.532 | 1.549964 | 5.691866 | 0.007355 | (M+Na)+ |
| 12 | Apiin | 565.1512 | 187.143 | 1.064007 | 68.84733 | 0.008206 | (M+H)+ |
| 13 | LysoPC(18:0) | 524.3692 | 164.729 | 9.724237 | 0.299018 | 0.009023 | (M+H)+ |
| 14 | 24,25-Hydroxyvitamin D3 | 381.3135 | 99.861 | 3.881449 | 0.438407 | 0.009608 | (M+H-2H2O)+ |
| 15 | Vitexin | 433.1119 | 428.4725 | 2.536539 | 4.011205 | 0.010521 | (M+H)+ |
| 16 | LysoPC(16:0) | 496.3357 | 60.74 | 1.467576 | 0.329862 | 0.011502 | (M+H)+ |
| 17 | Creatinine | 114.066 | 179.192 | 7.142859 | 2.679841 | 0.018955 | (M+H)+ |
| 18 | Pantothenate | 220.1172 | 276.944 | 1.752953 | 2.010937 | 0.023097 | (M+H)+ |
| 19 | 1-O-Octadecyl-sn-glyceryl-3-phosphorylcholine | 510.386 | 201.195 | 1.053211 | 0.441505 | 0.027515 | (M+H)+ |
| 20 | LysoPE(16:0/0:0) | 454.2903 | 202.938 | 2.638826 | 0.501703 | 0.031324 | (M+H)+ |
| 21 | PC(18:0/18:1(9Z)) | 810.596 | 146.074 | 3.194967 | 0.36285 | 0.04029 | (M+Na)+ |
| 22 | Daidzin | 461.0773 | 220.0965 | 1.094667 | 171.8161 | 0.040852 | (M-H+2Na)+ |
| 23 | Cholic acid | 373.2711 | 259.3995 | 2.411499 | 0.18822 | 0.043748 | (M+H-2H2O)+ |
| 24 | 2-Amino-2-methyl-1,3-propanediol | 70.06492 | 323.985 | 2.288642 | 3.486068 | 0.04661 | (M+H-2H2O)+ |
| 25 | 5-Aminopentanoic acid | 118.0857 | 396.965 | 2.87072 | 0.643758 | 0.046728 | (M+H)+ |
| 26 | Senecioic acid | 101.0594 | 396.881 | 2.286999 | 0.635643 | 0.048059 | (M+H)+ |
| 27 | Homoveratric acid | 195.0649 | 150.362 | 1.217428 | 79.77007 | 0.000185 | (M-H)- |
| 28 | Docosahexaenoic acid | 327.2299 | 42.9895 | 1.819638 | 0.160182 | 0.001104 | (M-H)- |
| 29 | Docosapentaenoic acid | 329.2466 | 40.018 | 3.626001 | 0.221912 | 0.002242 | (M-H)- |
| 30 | Isobutyric acid | 87.04579 | 119.756 | 2.49174 | 1.616909 | 0.005591 | (M-H)- |
| 31 | Propionic acid | 73.0297 | 171.381 | 1.730928 | 3.202493 | 0.015433 | (M-H)- |
| 32 | 3-Phenylpropanoic acid | 149.0603 | 103.3285 | 6.666644 | 0.048764 | 0.016559 | (M-H)- |
| 33 | 1-Palmitoyl-2-oleoyl-phosphatidylglycerol | 747.5129 | 39.3385 | 1.146691 | 0.351741 | 0.025876 | (M-H)- |
| 34 | Adynerin | 515.3007 | 52.1985 | 3.887747 | 0.454613 | 0.039544 | (M-H)- |
| 35 | Deoxycholic acid | 391.2846 | 154.143 | 16.81248 | 0.049599 | 0.045912 | (M-H)- |
